# Novel substance, same old problems: admissions of psychosis precipitated by hexahydrocannabinol, a widely available semi-synthetic cannabinoid

**DOI:** 10.1192/bjb.2025.85

**Published:** 2026-04

**Authors:** Brian O’Mahony, Sarah Lanigan, Níall Lally, Andrew O’Malley, Bobby Smyth, Colm McDonald, Brian Hallahan

**Affiliations:** 1 School of Medicine, University of Galway, Galway, Ireland; 2 Department of Psychiatry, University Hospital Galway, Galway, Ireland; 3 Department Public of Health & Primary Care, Trinity College Dublin, Dublin, Ireland

**Keywords:** Psychoses, substance-induced cannabinol/analogues and derivatives, psychoactive drugs, legal psychiatry

## Abstract

**Aims and method:**

To investigate the impact of the widespread availability and use of the semi-synthetic cannabinoid hexahydrocannabinol (HHC) on hospital admissions owing to psychosis. Medical records of patients admitted for psychotic illness to University Hospital Galway were examined to assess HHC or other illicit drug use before admission.

**Results:**

Of the 214 total admissions for psychotic illness, 28 admissions (13.1%) were preceded by use of HHC, nine of whom used only HHC. Sixteen (34%) admissions of first-episode psychosis were preceded by HHC use, with seven of these using only HHC.

**Clinical implications:**

Clinicians should explicitly enquire about the use of HHC in patients presenting with first-episode and relapse of psychotic illness. Sufficient evidence has now accumulated from research of a link between HHC and psychosis. Such psychoses appear to be more prominent in young people with early-phase psychosis, and highlights the need for authorities to change legislation to avoid further harm.

In the Republic of Ireland, ‘cannabis and products or preparations extracted from the cannabis plant which are psychoactive’ are listed as Schedule 1 substances, under the Misuse of Drugs Act, 1977.^1^ This classification renders the manufacture, production, preparation, sale, supply, distribution and possession of these products unlawful, except for research purposes. This Act specifically cites only ‘tetrahydro derivatives of cannabinol’ as the psychoactive component of the cannabis plant. This refers to delta-9-tetrahydrocannabinol (Δ9-THC), the main psychoactive compound of cannabis.^2^ The classification of cannabis as a Schedule 1 substance is driven by a variety of cultural and health factors, but a primary concern is the elevated risk of psychosis in people who consume these substances.^3^ This risk appears to increase in products with higher concentration of Δ9-THC,^4^ and appears to be higher still in products containing synthetic cannabinoids.^5^

Hexahydrocannabinol (HHC) is a semi-synthetic cannabinoid derived from cannabidiol (CBD), which is similar in chemical structure to Δ9-THC. It is classified as semi-synthetic, rather than synthetic, because it is synthesised from CBD, which is often sourced from low-THC cannabis (hemp). This terminology has been criticised as misleading, with one group proposing ‘Derived Cannabis Product’ as a more appropriate classification.^6^ The term semi-synthetic differentiates it from fully synthetic cannabinoids that are synthesised *de novo* and do not structurally resemble Δ9-THC or CBD. Its subjective psychoactive effects on humans have yet to be thoroughly investigated, with individuals in a Canadian online survey reporting psychoactive effects such as relaxation and euphoria.^7^ A laboratory study of two participants showing mild cannabimimetic effects after a participant vaped 15 mg of HHC without serious impairment, and no noticeable effects in a second participant who orally ingested of 20 mg of HHC.^8^ However, animal models indicate that it has a similar effect profile to Δ9-THC,^9,10^ with a similar receptor-biding profile also noted.^11^

HHC emerged in Europe in the autumn of 2022, gaining popularity as a semi-synthetic cannabinoid alternative to THC, and its proliferation across the continent led to close monitoring by the European Union Drugs Agency (EUDA).^11^ Beyond monitoring, the EUDA has failed to give any direction to countries in Europe regarding their legislative or regulatory response to HHC. In France, the first reported case of HHC intoxication occurred in October 2022, and sale of HHC was made illegal in June 2023.^11^ Similar legislative action was subsequently enacted in several other European countries.^11^ A paper from French Poison Centres reported a variety of harmful effects of HHC use and indicated an increasing use of HHC before the legislative change.^12^ Notably, this paper was first submitted in July 2023 and published in March 2024, indicating the difficulty in rapidly disseminating research about novel psychoactive substances.

We have previously reported on two cases of psychosis that appear to have been precipitated by use of HHC,^13^ and suggested that the sale of HHC fulfilled criteria for an illegal substance through use of the Criminal Justice (Psychoactive Substances) Act 2010 (PSA).^14^ This Act was introduced in Ireland in response to increased sales of novel psychoactive substances by so called ‘head shops’, and to reverse Ireland’s status as the heaviest user of novel psychoactive substances in Europe.^15^ Such substances led to a deleterious impact on the mental health of many individuals, including precipitating psychotic symptoms.^16,17^ This ‘catch-all’ banning of novel psychoactive substances was introduced as previous attempts to prohibit specific substances (including synthetic cannabinoids) through legislation had led to these head shops replacing sales of named substances with unnamed novel psychoactive substances, e.g. a ban on the sale of mephedrone led to the sale of flourotropococaine.^17^ Currently, HHC is marketed openly as a ‘legal’ alternative to cannabis products.^11^ It has been produced and marketed in various forms, including sprayed onto low-THC cannabis flower and resin, vaping cartridges and food products such as sweets. Irish retailers of CBD-related products, including HHC, are easily accessible online or via retail stores in most large urban centres, where its psychoactive effects are openly advertised.

Given the limited scope of our case series, we wished to quantify the prevalence of recent HHC usage in patients admitted to an acute psychiatric in-patient unit with an acute psychotic episode.

## Method

We carried out a retrospective review of medical notes of individuals admitted with acute psychotic illness to the Adult Acute Mental Health Unit, University Hospital Galway (AAMHU), over a 21-month period (May 2023 to December 2024). All patient data were anonymised, and all participant data stored securely and handled in accordance with the Data Protection Act 2018. Ethical approval was attained before study commencement from the Galway University Hospitals Research Ethics Committee (C.A. 3250). The authors assert that all procedures contributing to this work comply with the ethical standards of the relevant national and institutional committee on human experimentation with the Helsinki Declaration of 1975, as revised in 2013.

Patients were identified through use of an electronic database that tracks each admission, giving their initial and discharge diagnosis. Admissions were assessed as being preceded by HHC or other illicit drug use if their medical records indicated they had used these substances in the 2 weeks before their admission, or a urine drug screen performed on admission indicated substance use.

During the admission to the AAMHU, patients are asked about drug use, illicit or otherwise. All patients are asked to provide urine samples for dipstick drug screening, although this is not strictly enforced. This urine-drug analysis flags for recent use of cocaine, amphetamines, THC, opioids, benzodiazepines, barbiturates, methamphetamine, MDMA, tricyclic antidepressants and paracetamol. The structural similarity of HHC to Δ9-THC means that HHC and its metabolites can cross-react with standard THC immunoassays, potentially leading to false-positive results.^18,19^ As such, immunoassay techniques may not reliably distinguish between the two. Accurate identification of HHC therefore requires confirmatory testing using more specific analytical techniques, such as gas chromatography–mass spectrometry or liquid chromatography–mass spectrometry. The urine drug screens in University Hospital Galway did not make use of these more specific analytical techniques.

Inclusion criteria required patients to be over 18 years old and admitted for an acute psychotic illness, with the medical notes indicating a diagnosis corresponding to ICD-11 categories 6A20–25, 6C40.6–4E.6, 6A60.1, 6A70.4 or 6A71.4; indicating a psychotic disorder. From the patients’ electronic healthcare records, we collected information pertaining to the patients’ age and gender, any documented self-reported use of HHC or illicit psychoactive substances, and whether the given episode was the first episode of psychosis.

The Statistical Package for Social Sciences, Version 27.0 for Windows (SPSS 24, SPSS Inc., IBM, New York, USA) was utilised for data analysis. Descriptive statistics including means, standard deviations, frequencies and percentages were utilised for all categorical and continuous variables. Mann–Whitney *U*-test was used for non-parametric continuous data (i.e. age), with chi-squared or Fisher’s exact test utilised as appropriate for non-parametric dichotomous data (gender and HHC use).

## Results

**Fig. 1:**
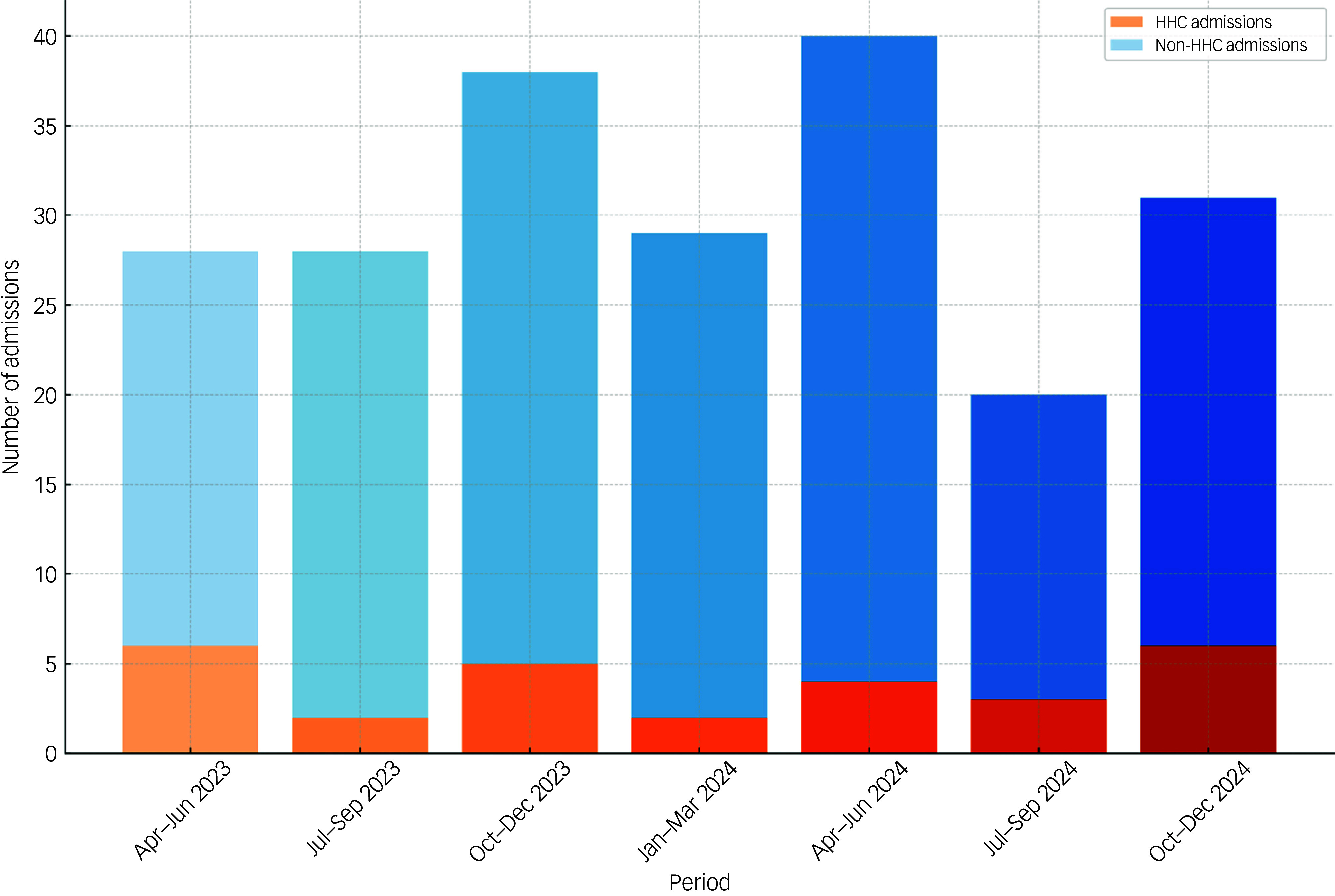
Per-quarter admissions of psychotic illness, and those preceded by hexahydrocannabinol use. HHC, hexahydrocannabinol.

We identified 214 total admissions of 173 individuals during the study timeframe. Among these, 137 (64%) were men, with the median age being 43 years (range 18–83 years). Of these 214 admissions, 28 (13.1%) were preceded by self-reported use of HHC. Among this cohort of admissions owing to psychosis, more HHC users were male (*n* = 22, 78.6%) than female (*n* = 6, 21.4%) (*χ* ^2^ = 9.14, *P* = 0.002). HHC users had a median age of 24 years (range = 18–49 years), which was significantly younger than non-users’ median age of 45 years (*U* = 130.5, *P* = 0.002). Figure 1 shows the rate of HHC admissions per month over the recorded period. Of these 28 admissions, nine used only HHC, with the other participants additionally using other psychoactive substances (cannabis *n* = 17, cocaine *n* = 7, psychedelics *n* = 2).

Of the total 214 admissions, 43 were documented to have used other illicit substances, but not using HHC. Table 1 displays the number of psychotic disorder diagnoses, and their associated preceding substance use. The most used illicit substance was cannabis (*n* = 32), followed by cocaine (*n* = 11) and psychedelics (*n* = 3).

**Table 1:** Diagnoses of psychosis and their associated preceding substance use

Diagnosis	*n*	Cannabis *n* (%)	Hexahydrocannabinol *n* (%)	Other drug use *n* (%)	Any drug use *n* (%)
Affective psychosis^a^	44	2 (4.5)	4 (9.1)	4 (9.1)	7 (15.9)
Non-affective psychosis^b^	131	11 (8.4)	4 (3.1)	5 (3.8)	15 (11.5)
Drug-induced psychosis	39	19 (48.7)	20 (51.3)	5 (12.8)	39 (100)
All psychosis	214	32 (15)	28 (13.1)	14 (6.5)	58 (27.1)
First-episode psychosis	47	16 (34.0)	16 (34.0)	5 (10.6)	23 (48.9)
Relapse of psychosis	167	16 (9.6)	12 (7.2)	9 (5.4)	35 (21.0)

a. Includes bipolar affective disorder – manic episode with psychosis, severe depressive episode with psychosis.

b. Includes schizophrenia, schizoaffective disorder and delusional disorder.

Of the 173 individuals included in the study, 47 were admitted to the AAMHU during their first episode of psychosis. Of these 47 individuals, 16 (34%, 95% CI 20.9–49.3%) were preceded by HHC use, and seven (14.9%, 95% CI 6.2–28.3%) were preceded only by HHC use.

## Discussion

To our knowledge, this is the first study to examine prevalence rates of HHC use in individuals experiencing acute psychotic disorders. Our findings highlight high prevalence of HHC use among individuals admitted with psychotic symptoms, particularly first-episode psychosis cases (over a third). HHC is now the second most common drug involved in psychosis presentations. The results show that rates of such admissions have stayed relatively stable over the recorded 21-month period.

These findings align with prior data when psychoactive substances including synthetic cannabinoids and amphetamine-like substances were available legally in head shops, which showed that nearly 13% of mental health patients consumed such substances, with 74% of these using synthetic cannabinoids.^20^

Indeed, it is probable that this limited retrospective study underestimates the prevalence of HHC use in the study population. HHC use may result in a urine toxicology giving a false positive result for THC, or go undetected, depending on the individual test kit used.^11^ Additionally, a lack of awareness among staff regarding the prevalence of this novel substance and its potential to induce psychosis likely resulted in patients not being directly asked about their use of HHC. Similarly, when asked a screening question about illicit or illegal drug use, patients may truthfully have answered no, as HHC is not an illegal substance; and the topic may consequently not have been investigated further.

HHC, through its R epimer, has a similar binding profile to the CB1 receptor compared with Δ9-THC.^21^ HHC’s potential to induce psychosis likely stems from its binding to the endocannabinoid CB1 and CB2 receptors in a similar fashion to Δ9-THC, the main psychoactive component of cannabis. Agonism of the CB1 receptor leads to dopamine release in the nucleus accumbens and ventral tegmental area.^22^ Acute Δ9-THC ingestion leads to increased dopamine release and neuronal activity in the striatum,^23^ and long-term use is associated with blunting of the dopamine system.^24–26^ HHC is therefore likely to produce similar stimulation of dopamine release in areas such as the medial temporal lobe and striatum, areas high in CB1 expression and whose activity levels are correlated with psychotic symptoms.^27^

The ease of access to novel psychoactive substances and their potential role in rising psychotic illnesses in Ireland is not a new phenomenon. This is not to suggest that users of novel psychoactive substances consume these drugs with reckless disregard for their consequences. Users may assume that commercially available products are safe and well tested.^28,29^ The widespread sale of novel psychoactive substances in Ireland, before the introduction of the PSA, appeared to have been linked to an increasing number of drug-related psychiatric admissions, with the Act’s introduction reversing this trend.^17^ The Act was deliberately broad in its definition of psychoactive substance (Box 1).

Box 1 Irish Criminal Justice (Psychoactive Substances) Act definition of a psychoactive substance

a substance, product, preparation, plant, fungus or natural organism which has, when consumed by a person, the capacity to –

(a) produce stimulation or depression of the central nervous system of the person, resulting in hallucinations or a significant disturbance in, or significant change to, motor function, thinking, behaviour, perception, awareness or mood,

or

(b) cause a state of dependence, including physical or psychological addiction

The PSA was the first of its kind in the world, and although it was criticised as legally and psychopharmacologically unsound, very similar legislation (Psychoactive Substances Act 2016) was subsequently introduced in the UK and elsewhere.^30,31^ In an interesting contrast to Ireland, HHC is not widely available in the UK as its sale is deemed illegal under this Act. Despite proceedings in Dáil Éireann (the upper chamber of the Irish parliament) indicating that the sale of HHC, ‘which one would assume is for human consumption, is guilty of an offence’ under the PSA, HHC continues to be widely sold across the Republic of Ireland.

In March 2025, HHC was placed under international control by the United Nations Commission on Narcotic Drugs (CND), following a recommendation by the World Health Organization Expert Committee on Drug Dependence.^32^ Ireland will now be obliged to ban HHC under the Misuse of Drugs Act, although such a ban will have come 2 years after HHC was banned in France, and over a year since concerns about its harmful psychoactive effects were first raised by Irish psychiatrists.^13^ This raises concerns about the ability of the PSA to prevent the sale of future novel psychoactive substances. Bans on HHC in other jurisdictions have previously been followed by increased sales of novel psychoactive substances, such as HHC-O and THC-P.^18^ Another concern is that the CND’s addition of HHC to the list of prohibited substances may have unintended consequences. It is possible that people who sell and use HHC will stockpile it in advance of an Irish ban occurring, as occurred during the period of the head shops.^33^ This may inadvertently result in a spike in use and acute harms in the weeks after a ban.

Should sale and use of different novel psychoactive substances become widespread in Ireland, the failure to use the PSA to control sale of HHC paints a worrying picture. Classification of a substance as psychoactive appears to require a different avenue to scientific publications, with its associated time delays in ethics applications, manuscript submission and peer review. Our paper shows that the delay in banning HHC has led to significant harm for a number of individuals, and failure to react more quickly to the next novel psychoactive substances will likely cause future harm.

This study is associated with a number of limitations. First, we were unable to capture any individuals experiencing a first episode of psychosis who did not require admission to the acute psychiatric in-patient unit. Consequently, we only included individuals experiencing a severe or very acute psychotic episode, and not those who were managed for their psychotic episode in primary care or within secondary out-patient mental health services (i.e. day hospital, out-patient clinics). Second, given that all patients lived in one geographical region, its findings may not be generalisable, although this unit covers a mixture of rural and urban areas. Third, a key limitation is the absence of any confirmatory analysis of HHC for any of these admissions on a urine drug screen, as well as the fact that a majority of patients did not provide a urine drug sample. This is compounded by the observation that reliance on self-reporting of substance use likely vastly underestimates its extent.^34,35^ Lastly, this study took place in one hospital, and so the results may not be generalisable.

In conclusion, our results lend further support to the putative link between HHC use and development of psychotic illness. The scope of this study was limited, and future research in this area should involve direct enquiry regarding HHC use in individuals experiencing, or who have experienced, psychotic illness, including those treated as in-patients and out-patients who have experienced either a first episode or relapse of psychosis. Clinicians should explicitly enquire about HHC use in all patients undergoing treatment for a first episode or relapse of psychotic illness. Psychoeducation programmes for individuals with psychotic disorders regarding the risks of HHC are advisable. The public should be informed about the potential mental health risks associated with this product. The failure of the EUDA to provide clear advice to European governments on legislative responses to HHC is disappointing. Finally, this study adds weight to the view that HHC is indeed psychoactive as defined in the Criminal Justice (Psychoactive Substances) Act in the Republic of Ireland. As such, those involved in importation, distribution and sale of this drug appear to be operating in breach of this Act. Clinicians had been of the view that the Act constituted an important first line of defence in Ireland against open sale of psychoactive drugs. This appears to not be the case, and the years-long continued sale of HHC raises questions about the ability of this Act to guard against future novel psychoactive substances. This longstanding, tolerated and ongoing sale of HHC in Ireland has harmed many people. In preparing for the next iteration of the National Drugs Strategy in Ireland and across Europe, steps should be taken to ensure a more robust, proactive and nimble response to the next commercialised novel psychoactive substances, to better protect the health of citizens.

## Data Availability

The data that support the findings of this study are available from the corresponding author, B.O., upon reasonable request.

## About the authors

**Brian O’Mahony,** MB, BCh, BAO, MSc, is a senior registrar in psychiatry who worked at University Hospital Galway, and was a visiting scholar at the University of Galway at the time of writing this research. **Andrew O’Malley,** MB, BCh, BAO, is a senior registrar in psychiatry who worked at University Hospital Galway at the time of writing this research. **Bobby Smyth,** MB, BCh, BAO, PhD, is a consultant psychiatrist and was Clinical Professor of Public Health & Primary Care at Trinity College Dublin at the time of writing this research. **Niall Lally,** MB, BCh, BAO, PhD, is a registrar in psychiatry who worked at University Hospital Galway at the time of writing this research. **Sarah Lanigan,** MB, BCh, BAO, is a senior house officer in psychiatry who worked at University Hospital Galway at the time of writing this research. **Colm McDonald,** MB, BCH, BAO, MRCPsych, PhD, is a consultant psychiatrist with University Hospital Galway and Professor of Psychiatry at the University of Galway. **Brian Hallahan,** MB, BCH, BAO, MRCPsych, MD, is a consultant psychiatrist with University Hospital Galway and Senior Lecturer in Psychiatry at the University of Galway.
